# Data on PAGE analysis and MD simulation for the interaction of endonuclease Apn1 from *Saccharomyces cerevisiae* with DNA substrates containing 5,6-dihydrouracyl and 2-aminopurine

**DOI:** 10.1016/j.dib.2018.09.007

**Published:** 2018-09-12

**Authors:** Elena S. Dyakonova, Vladimir V. Koval, Alexander A. Lomzov, Alexander A. Ishchenko, Olga S. Fedorova

**Affiliations:** a Institute of Chemical Biology and Fundamental Medicine, Siberian Branch of the Russian Academy of Sciences, 8 Lavrentyev Ave., Novosibirsk 630090, Russian Federation; b Department of Natural Sciences, Novosibirsk State University, 2 Pirogov St., Novosibirsk 630090, Russian Federation; c Groupe «Réparation de l׳ADN», Equipe Labellisée par la Ligue Nationale Contre le Cancer, CNRS UMR8200, Univ. Paris-Sud, Université Paris-Saclay, F-94805 Villejuif, France; d Gustave Roussy, Université Paris-Saclay, F-94805 Villejuif, France

## Abstract

This article presents new data on nucleotide incision repair (NIR) activity of apurinic/apyrimidinic endonuclease Apn1 of *Saccharomyces cerevisiae*, which is known as a key player of the base excision DNA repair (BER) pathway, see “Yeast structural gene (APN1) for the major apurinic endonuclease: homology to Escherichia coli endonuclease IV” [1], “Abasic sites in DNA: repair and biological consequences in Saccharomyces cerevisiae” [2] and “Characterisation of new substrate specificities of Escherichia coli and Saccharomyces cerevisiae AP endonucleases” [3]. The characterization of NIR activity of wild type Apn1 and mutant form Ape1 H83A were made by denaturing PAGE analysis, and MD simulations of Apn1 complexed with DNA containing 5,6-dihydro-2′-deoxyuridine (DHU) and 2-aminopurine (2-aPu) residues. This data article is associated to the manuscript titled “Apurinic/apyrimidinic endonuclease Apn1 from *Saccharomyces cerevisiae* is recruited to the nucleotide incision repair pathway: kinetic and structural features” [4].

*Published by Elsevier Inc. This is an open access article under the CC BY license (*http://creativecommons.org/licenses/by/4.0/*).*

**Specifications table**

**Table:** 

Subject area	Biochemistry
More specific subject area	Structural enzymology, enzymatic catalysis
Type of data	Text file, graph, autoradiograph, figure, movie
How data was acquired	Data was obtained using PAGE assay, stopped-flow technique, nonlinear regression fitting and MD simulation
Data format	Analyzed data
Experimental factors	Used DNA is 12mer duplex containing damaged nucleotide DHU or abasic site and fluorescent 2-aminopurine residue located upstream/downstream of damaged site
Experimental features	Interaction of WT or H83A Apn1 with substrate DNA was analyzed by denaturing 20% PAGE
	MD simulation was performed in the AMBER 14 MD modeling software with GPU accelerated code
Data source location	Institute of Chemical Biology and Fundamental Medicine of Siberian Branch of the Russian Academy of Sciences, 8 Lavrentyev Ave., Novosibirsk, 630090, Russian Federation
Data accessibility	Data are available with this article
Related research article	[4] E.S. Dyakonova, V.V. Koval, A.A. Lomzov, A.A. Ishchenko, O.S. Fedorova, The role of His-83 of yeast apurinic/apyrimidinic endonuclease Apn1 in catalytic incision of abasic sites in DNA, Biochim. Biophys. Acta 1850 (2015) 1297–1309, https://doi.org/10.1016/j.bbagen.2015.03.001

**Value of the data**

• The data of MD simulation provide information for the structures of WT Apn1 complexed with NIR substrates, containing 5,6-dihydrouracil and 2-aminopurine residues.
• The data illustrates that efficiency of NIR catalysis driven by Apn1 depends strongly on the spatial structure of DNA-substrates.
• The data could be useful guidelines for further design of new anti-fungal and anti-malarial agents as much as yeast Apn1 belongs to Endo IV family, which members are not found in mammalian cells, but are present in many microorganisms.

## 1 Data

Data reported here describe the features of nucleotide incision repair (NIR) of DNA catalyzed AP-endonuclease by Apn1 from *Saccharomyces cerevisiae* as revealed from kinetic studies and MD simulation analysis.

### 1.1 How is optimization of obtained data (kinetic traces) using stopped-flow technique executed?

**Fig. 1:**
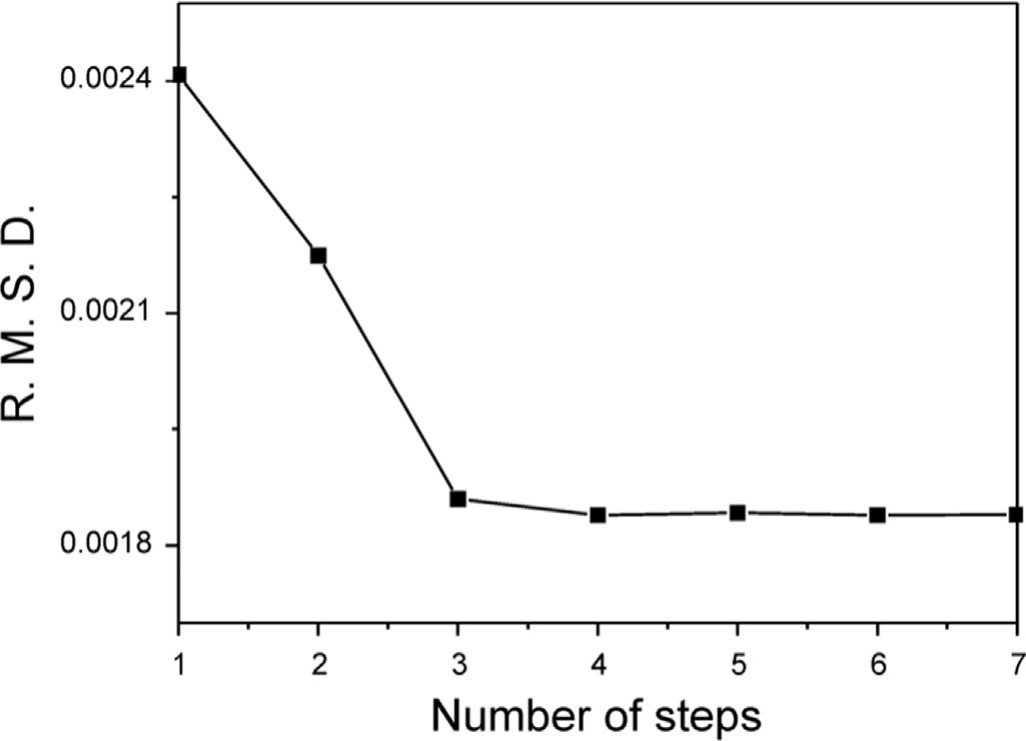
The scree test for the scheme describing WT Apn1 interaction with substrate ***DHU(2 aPu)*** in BER buffer. Oligodeoxyribonucleotide (ODN) duplex ***DHU(2-aPu)*** is 5′**-**d(CTCTC(DHU)(2**-**aPu)CTTCC)**-**3′ complemented with 5′**-**d(GGAAGCGGAGAG)**-**3′. Concentrations of WT Apn1 and ODNs were 2.0 and 1.5 μM, respectively. Root mean standard deviations (R.M.S.D.) of the residuals after fitting to an *n*-step binding model are plotted versus n. The number of steps corresponding to the beginning of the shallow-slope (scree) region appears to be the minimal number for adequately describing the binding.

**Scheme 1:**
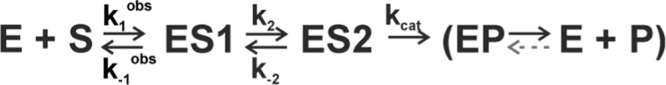
Kinetic scheme of the interaction of Apn1 with substrate ***DHU(2-aPu)***, containing two binding steps.

**Scheme 2:**
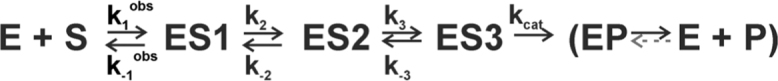
Kinetic scheme of the interaction of Apn1 with substrate ***DHU(2-aPu)***, containing three binding steps.

To optimize the kinetic scheme, which would be describe the kinetic traces obtained by stopped-flow technique [4], the proposed mechanisms should be examined by adding a gradual stage of the enzyme–substrate complex transformation, with replot and analysis of residuals being carried out. Global nonlinear least-squares fitting of the data obtained was performed in the DynaFit software (BioKin Ltd., USA) [5]. The scree test was conducted for validation of the proposed kinetic scheme (Fig. 1). Two- or three-step binding mechanisms describing Apn1׳s interaction with substrate ***DHU(2-aPu)*** in BER buffer are represented as Scheme 1, Scheme 2, respectively.

### 1.2 The influence of Mg2+ concentration

**Fig. 2:**
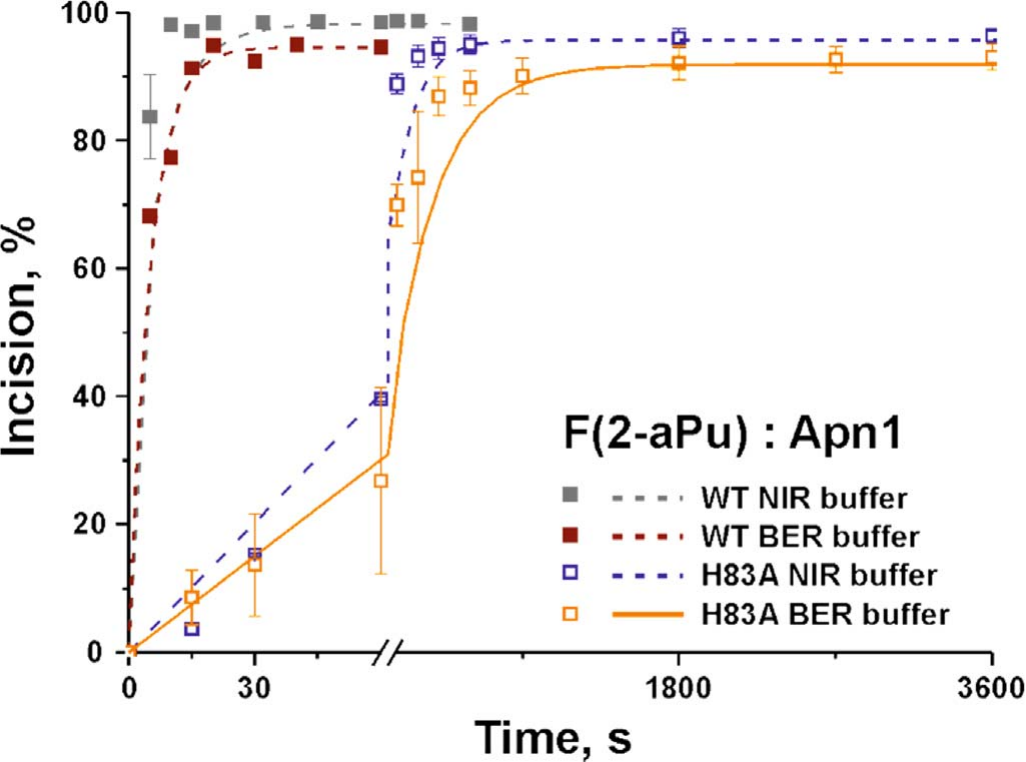
Incision of BER substrate *F(2-aPu)* by WT or H83A Apn1 in NIR buffer (20 mM HEPES-KOH pH 7.6, 50 mM KCl, 5 mM MgCl2, 1 mM DTT, 0.1 mM EDTA) or BER buffer (100 mM HEPES-KOH pH 7.6, 100 mM KCl). A pairwise comparison of catalytic incision in BER and NIR buffers between the following interactions: substrate *F(2-aPu)* with WT Apn1; substrate *F(2 aPu)* with H83A Apn1 (in all cases [*F(2-aPu)*] = [Apn1] = 1.5 μM). ODN substrate *F(2-aPu)* is 5′-d(CTCTCF(2-aPu)CTTCC)-3′ complemented with 5′-d(GGAAGCCGAGAG)-3′.

Dependence of AP endonuclease activities of WT or H83A Apn1 on Mg^2+^ ion concentration was tested using 12mer DNA duplexes containing tetrahydrofuran analog of AP site (F), and downstream mispaired 2-aPu residue. The main difference of NIR and BER buffers is 5 mM Mg^2+^ ions presence or absence, respectively (Fig. 2).

### 1.3 The assay of NIR activity of Apn1 wt AND and H73A in the case of DNA substrate containing 2-aminopurine upstream to DHU (2-aPu)DHU

**Fig. 3:**
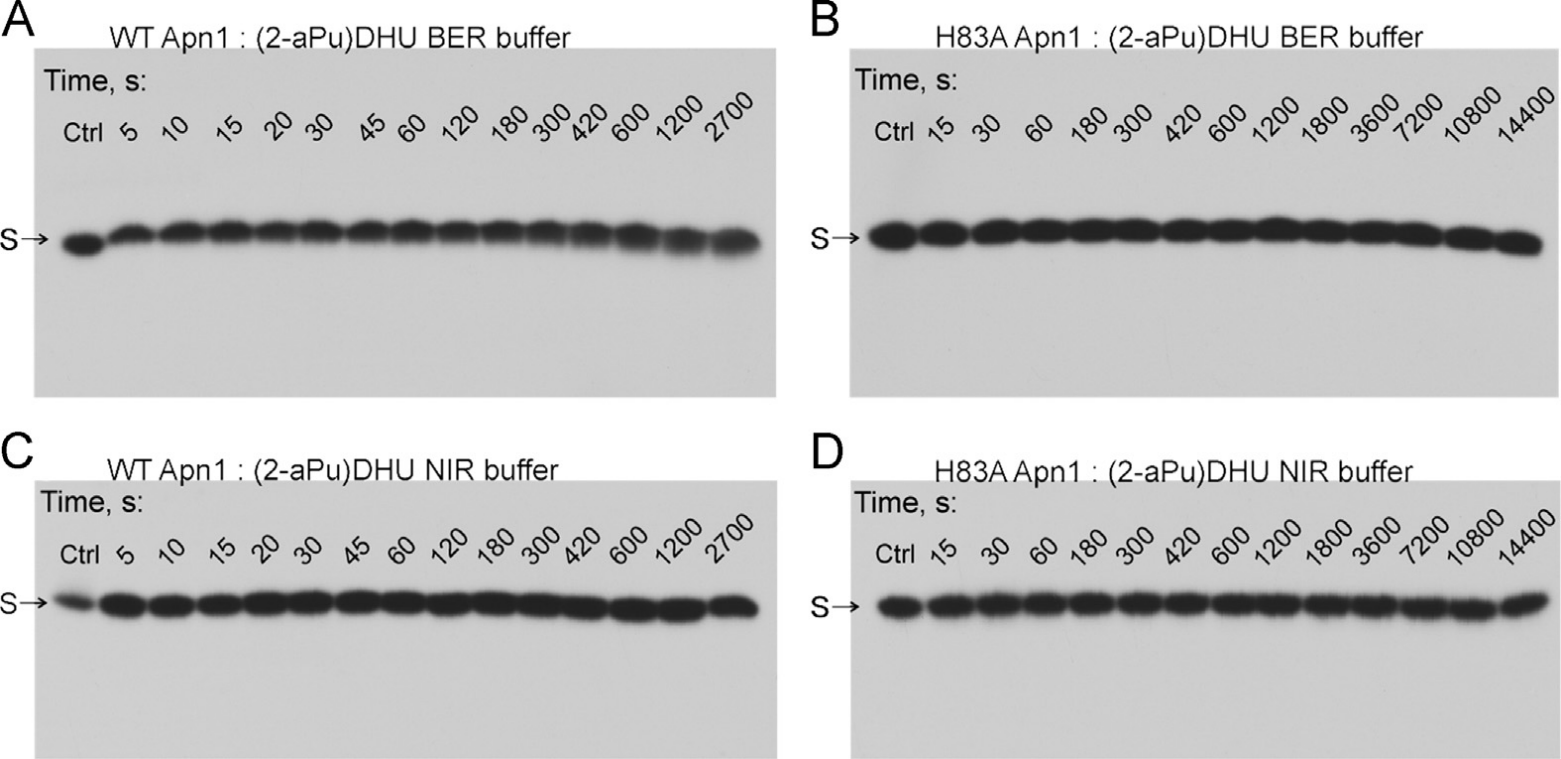
Time-dependent incision of the ^32^P-***(2-aPu)DHU*** duplex during the interaction with WT Apn1 (A) or H83A Apn1 (B) in BER buffer or WT Apn1 (C) or H83A Apn1 (D) in NIR buffer. S: ^32^P-***(2-aPu)DHU*** duplex. Analysis was conducted by denaturing PAGE in a 20% gel. Autoradiographs are shown.

PAGE assay of NIR activities of WT Apn1 or H83A Apn1 during the interaction with DNA duplex containing upstream 2-aminopurine residue of DHU (Fig. 3). Experiments were carried out in BER or NIR buffer. ODN duplex ***(2-aPu)DHU*** is 5′**-**d(CTCT(2**-**aPu)(DHU)CCTTCC)**-**3′ complemented with 5′**-**d(GGAAGGGCAGAG)**-**3′.

### 1.4 The influence of Zn^*2+*^ ion concentrations on interaction of Apn1 WT and H83A with (2-aPu)DHU

**Fig. 4:**
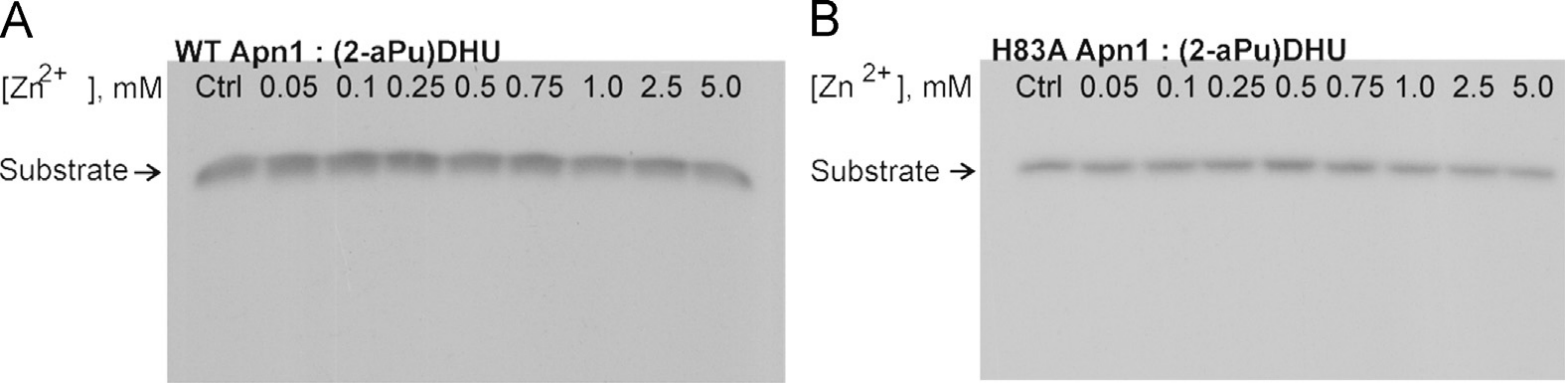
The effect of Zn^2+^ ion concentration on NIR activity of WT Apn1 (A) or H83A Apn1 (B) in a reaction with the ***(2-aPu)DHU*** duplex in BER buffer. Aliquots were taken 3 min after initiation of the reaction. Analysis was conducted by denaturing PAGE in a 20% gel. Autoradiographs are shown.

Experiments on reactivation of Apn1 forms during the interaction with substrate ***(2-aPu)DHU*** were conducted under different Zn^2+^ ion concentrations in the reaction solution (Fig. 4.).

### 1.5 Study of NIR activity of WT Apn1

**Fig. 5:**
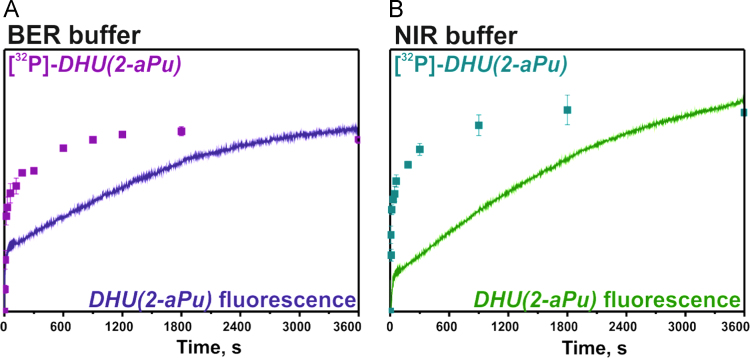
Interaction of substrate *DHU(2-aPu)* with WT Apn1 ([Apn1] = [*DHU(2-aPu)*] = 1.5 μM) presented as superimposition of a stopped-flow kinetic trace and product accumulation obtained by PAGE in BER buffer (A) or in NIR buffer (B). The kinetic trace obtained for *DHU(2-aPu)* by the stopped-flow technique is represented by the solid line, and that for ^32^P-*DHU(2-aPu)* obtained by PAGE is indicated with squares.

NIR activity of WT Apn1 was recorded by stopped-flow technique [4] (2-aPu fluorescence intensity detection) or monitored using denaturing PAGE (Fig. 5).

### 1.6 Molecular dynamics simulations of WT Apn1 complexed with DNA containing DHU

**Fig. 6:**
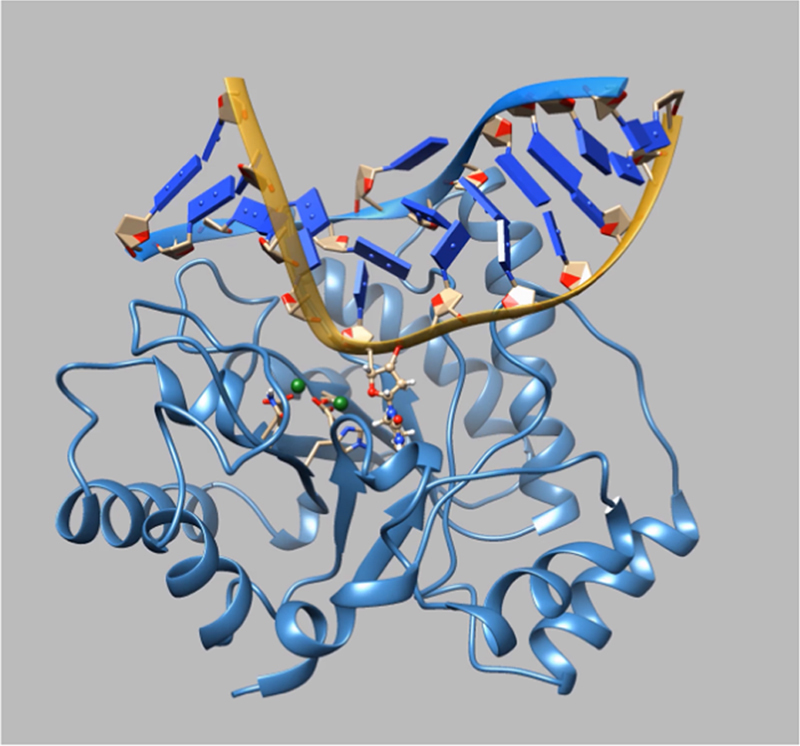
The MD movie for WT Apn1 complexed with substrate ***DHU*** was captured during 45 ns trajectory playback (https://drive.google.com/file/d/1FLC3Ns0fWR52w3BKW9XHxCUF9sbrSmzp/view?usp=sharing).

**Fig. 7:**
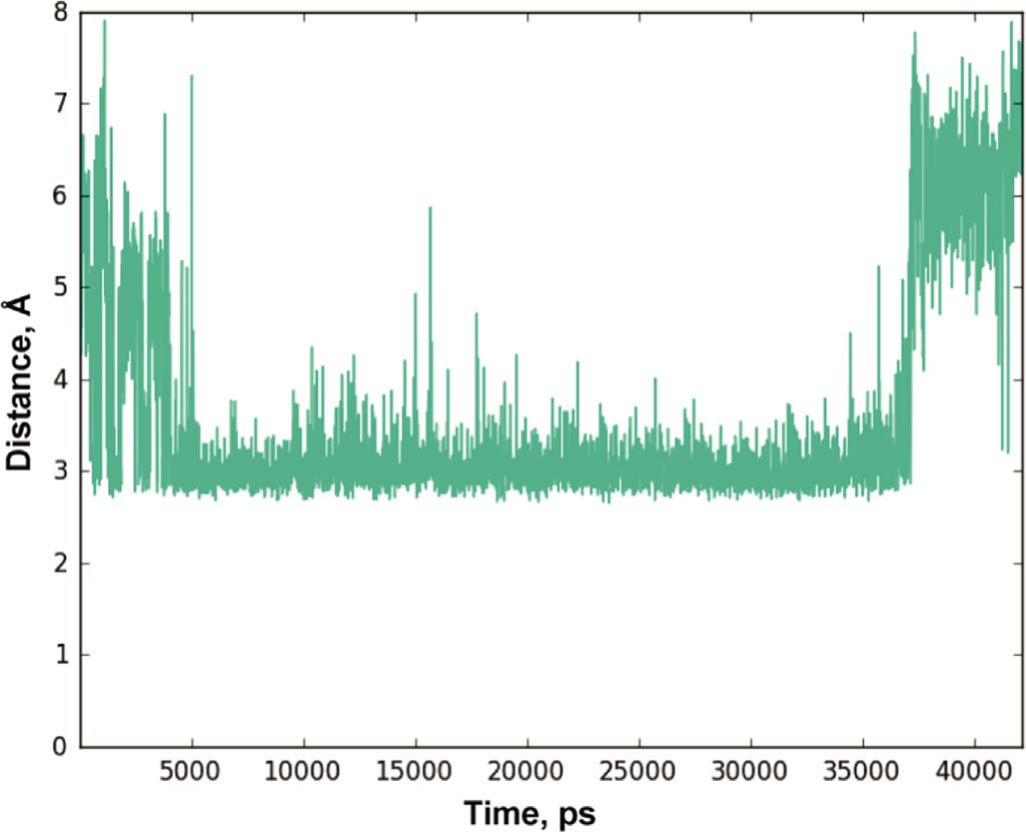
Distance changes between the N3 atom of the DHU residue and the side chain oxygen of Asn-279 in molecular complex Apn1–***DHU***. Duration of the MD simulation is 45 ns.

**Fig. 8:**
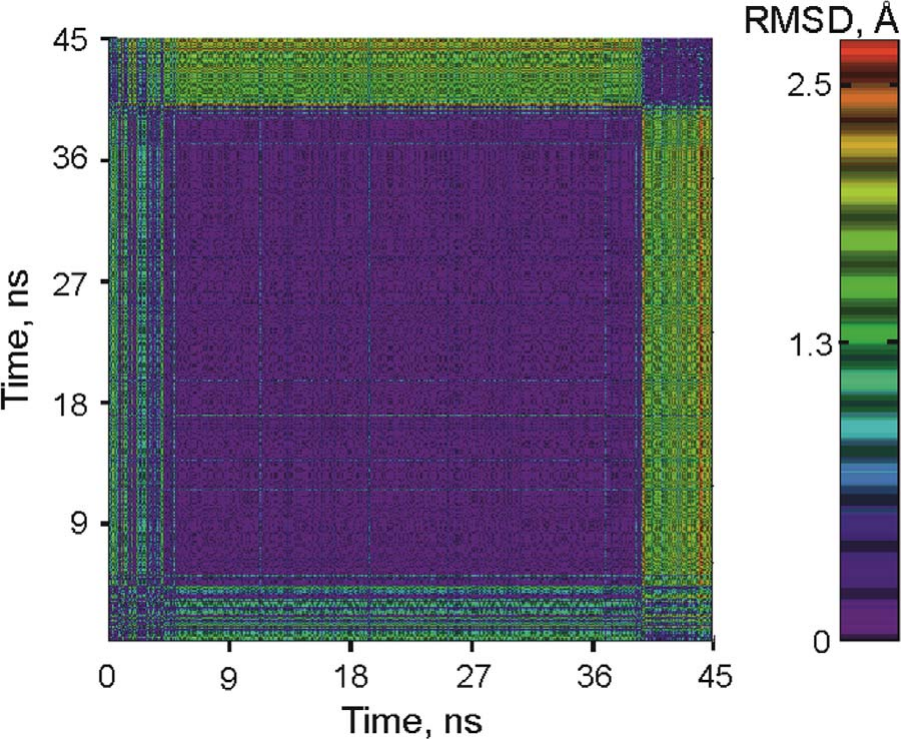
General characteristics of MD simulations of Apn1. A 2D R.M.S.D. plot of values calculated along a 45 ns trajectory segment for the complex of Apn1 with DHU-containing DNA with three Zn^2+^ ions. The x- and y-axes denote MD simulation time in ns.

**Fig. 9:**
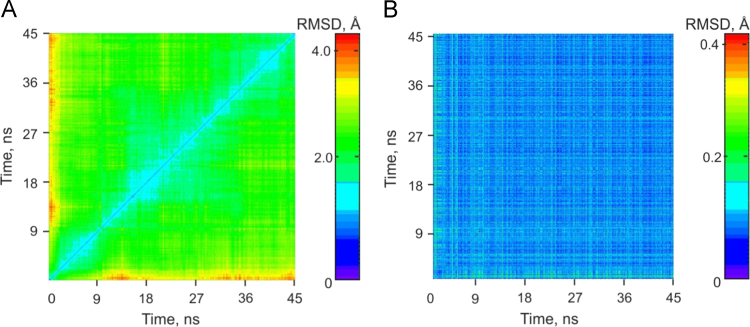
General characteristics of MD simulations of Apn1 complexed with one of DHU-DNA oligos containing a 2-aPu residue. A 2D R.M.S.D. plot of values calculated along a 45 ns trajectory for Apn1 with the ***DHU(2-aPu)*** duplex (A) or ***(2-aPu)DHU*** duplex (B). The x- and y-axes represent the MD simulation time in nanoseconds.

In this MD simulation, a WT Apn1 molecule contained three Zn^2+^ ions and was complexed with duplex ***DHU***. Oligodeoxyribonucleotide duplex ***DHU*** is 5′**-**d(CTCTC(DHU)CCTTCC)**-**3′ complemented with 5′**-**d(GGAAGGGGAGAG)**-**3′. Fig. 6 demonstrates MD movie for WT Apn1 complexed with substrate ***DHU***. In Fig. 7 distance changes between the N3 atom of the DHU residue and the side chain oxygen of Asn-279 in molecular complex Apn1–***DHU*** during 45 ns MD simulation are presented. General characteristics of MD simulations of Apn1 complexed with the ***DHU***, ***DHU(2-aPu)*** or ***(2-aPu)DHU*** duplex are illustrated in Fig. 8, Fig. 9.

Supplementary material related to this article can be found online at https://doi.org/10.1016/j.dib.2018.09.007.

The following is the Supplementary material related to this article Movie 1.

Movie 1 The MD movie for WT Apn1 complexed with substrate DHU was captured during 45 ns trajectory playback..

**Fig. 10:**
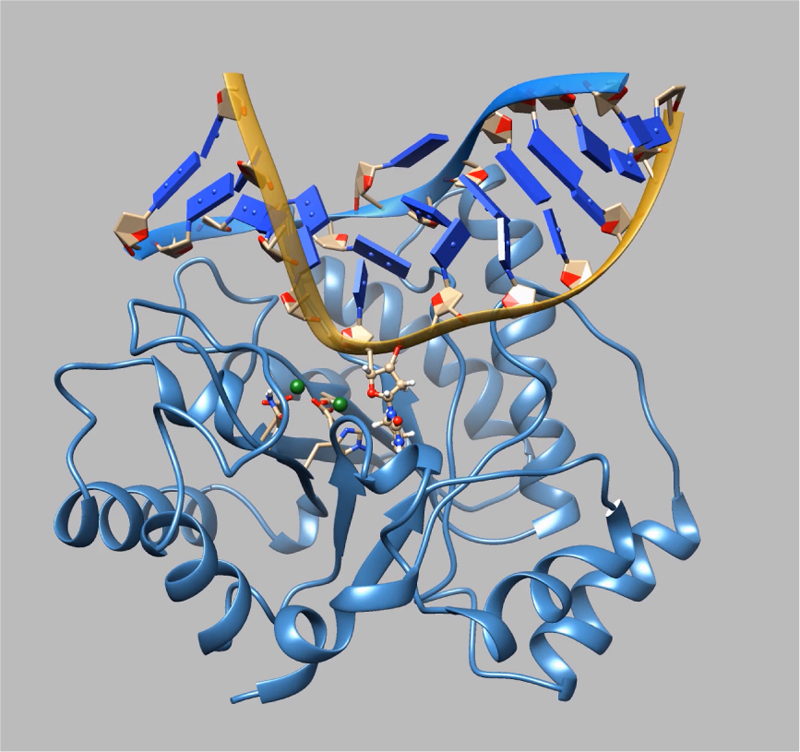
The MD movie for Apn1 with the ***DHU(2-aPu)*** duplex was captured during 45 ns trajectory playback (https://drive.google.com/file/d/1dqEsuqUZYiEo87KldOqqET9hZ3gRXA_O/view?usp=sharing). The 2-aPu residue (red ball-and-stick representation) is downstream of DHU.

**Fig. 11:**
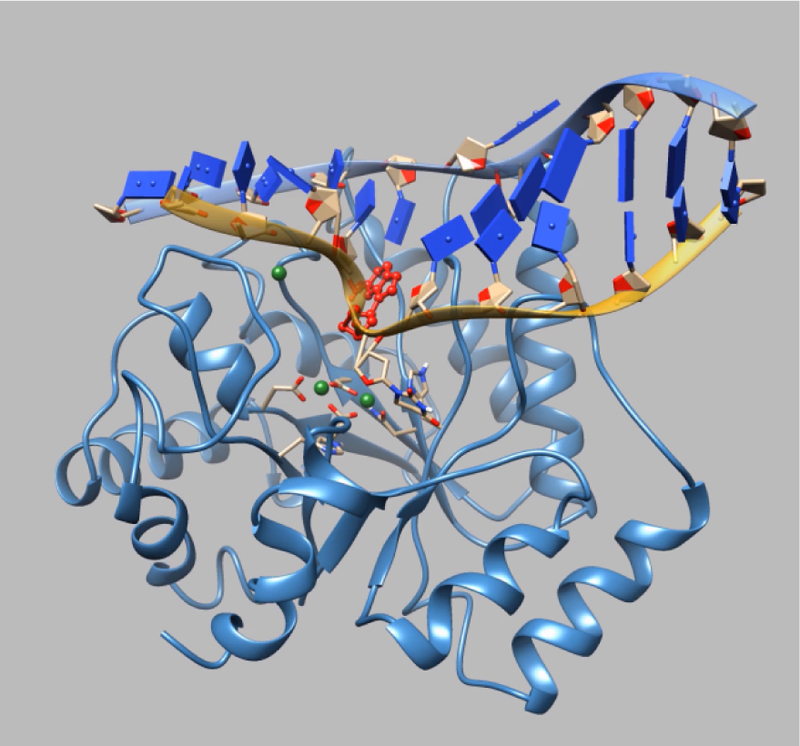
The MD movie for Apn1 with the ***(2-aPu)DHU*** duplex was captured during 45 ns trajectory playback (https://drive.google.com/file/d/1URgiMaaz_CmrcwSwyajSNSnkgk8lE6ZZ/view?usp=sharing). The 2-aPu residue (red ball-and-stick representation) is upstream of DHU.

MD movies for WT Apn1 complexed with substrates ***DHU(2-aPu)*** (5′**-**d(CTCTC(DHU) (2**-**aPu)CTTCC)**-**3′ complemented with 5′**-**d(GGAAGCGCAGAG)**-**3′) or ***(2-aPu)DHU*** (5′**-**d(CTCT(2**-**aPu)(DHU)CCTTCC)**-**3′ complemented with 5′**-**d(GGAAGGGCAGAG)**-**3′) are presented in Fig. 10, Fig. 11, respectively.

Supplementary material related to this article can be found online at https://doi.org/10.1016/j.dib.2018.09.007.

The following is the Supplementary material related to this article Movie 2, Movie 3.

Movie 2 The MD movie for Apn1 with the DHU(2-aPu) duplex was captured during 45 ns trajectory playback. The 2-aPu residue (red ball-and-stick representation) is located downstream of DHU.

Movie 3 The MD movie for Apn1 with the (2-aPu)DHU duplex was captured during 45 ns trajectory playback. The 2-aPu residue (red ball-and-stick representation) is located upstream of DHU..

## 2 Experimental design, materials and methods

### 2.1 S. cerevisiae WT and H83A Apn1 and DNA-substrates

Expression and purification of wild type (WT) Apn1 and mutant form H83A Apn1 were carried out as previously described [6], [7], [8].

Oligodeoxyribonucleotide (ODN) duplexes used as DNA-substrates were synthesized and purified according to [6], [7].

### 2.2 Kinetic data analysis

Global nonlinear least-squares kinetic analysis was performed in the DynaFit software (BioKin Ltd., USA) [5] as described in [9], [10].

### 2.3 An incision assay

The DNA cleavage kinetics *in vitro* conditions was studied using electrophoresis in polyacrylamide gel (PAGE) as described previously [6], [7]. The measurements were conducted at 25 °C in BER or NIR reaction buffer (BER buffer: 100 mM 4-(2-hydroxyethyl)-1-piperazineethanesulfonic acid (HEPES)-KOH (pH 7.6), 100 mM KCl; NIR buffer: 20 mM HEPES-KOH (pH = 7.6), 50 mM KCl, 0.1 mg/mL BSA, 1 mM DTT, 5 mM MgCl_2_).

### 2.4 MD simulations

The initial structure of a DNA duplex (PDB ID: 2NQJ [11]) was manually truncated to a 12mer and edited according to a nucleotide sequence being studied containing 2-aPu and/or DHU residues. Zn^2+^ ions were placed in the PDB file according to refs. [12], [13] and the data obtained on the CheckMyMetal server and RaptorX-Binding server [14]. Parameterization of Zn^2+^ ions in a protein for MD simulations remains a challenge with classical mechanics. In this work, we tested different approaches to Zn^2+^ parameterization: the cationic dummy atom (CaDA) approach [15] that involves virtual atoms to impose an orientational requirement for zinc ligands; the polarizable atomic multipole-based electrostatic model [16]; and the classic nonbonded atom method [17]. Finally, we found that the nonbonded atom method is more suitable for our purposes; accordingly, in this work, we chose this approach. Parameterization of Zn^2+^ ions was carried out as in ref. [17]. Structure refinement and molecular dynamic simulation were performed as in [7] using AMBER 14 molecular modeling suite [18], [19]. The force field parameters for the 2-aminopurine-5′-phosphate residue were retrieved from ref. [20]. The partial atom charges and force fields for the DHU residue were custom-parameterized calculated by the RESP method [21] based on the quantum mechanical calculation in the HF/6–31 G* using Gaussian’09 software [22]. A 45 ns MD simulation was conducted using the AMBER 14 GPU-accelerated code [18], [23] by means of the ff99SB force field [24], [25].Molecular graphics, MD movie generation, and trajectory analysis were carried out in the UCSF Chimera software [26].

## Transparency document Supporting information

Transparency data associated with this article can be found in the online version at 10.1016/j.dib.2018.09.007.

### Transparency documentSupplementary material

Supplementary material

.
