# Supplementary material for: Data on PAGE analysis and MD simulation for the interaction of endonuclease Apn1 from Saccharomyces cerevisiae with DNA substrates containing 5,6-dihydrouracyl and 2-aminopurine
Source: Data Brief. 2018 Sep 12;20:1515–24. doi: 10.1016/j.dib.2018.09.007 (PMC6334592; doi:10.1016/j.dib.2018.09.007)
Supplement: Supplementary file 4 — Supplementary material [file mmc1.pdf]

Data in Brief

Conflict of Interest Policy

Manuscript number (if applicable):
Article Title: Data on PAGE analysis and MD simulation for the interaction of endonuclease Apn1 from *Saccharomyces cerevisiae* with DNA substrates containing modified bases 5,6-dihydrouracil and 2-aminopurine

Author name:
Prof. Olga S. Fedorova

Declarations

Conflict of Interest

A conflicting interest exists when professional judgement concerning a primary interest (such as patient's welfare or the validity of research) may be influenced by a secondary interest (such as financial gain or personal rivalry). It may arise for the authors when they have financial interest that may influence their interpretation of their results or those of others. Examples of potential conflicts of interest include employment, consultancies, stock ownership, honoraria, paid expert testimony, patent applications/registrations, and grants or other funding.

Please state any competing interests

No

Funding Source

All sources of funding should also be acknowledged and you should declare any involvement of study sponsors in the study design; collection, analysis and interpretation of data; the writing of the manuscript; the decision to submit the manuscript for publication. If the study sponsors had no such involvement, this should be stated.

Please state any sources of funding for your research

This work was supported by the Federal Agency of Scientific Organizations (VI.57.1.2, 0309-2016-0001) to O.S.F., grants from RFBR (16-04-00037) to O.S.F. and RFBR (18-04-00596) to V.V.K., the Russian Ministry of Education and Science (NSU-SB RAS Collaborative Lab) under 5-100 Excellence Programme to V.V.K., Equipe LNCC 2016 and PRC CNRS/RFBR n1074 REDOBER to A.A.I.. The funders had no role in study design, data collection and analysis, decision to publish, or preparation of the manuscript.

Signature (a scanned signature is acceptable)

Prof. Olga S. Fedorova
